# Short-Chain Inulin Modulates the Cecal Microbiota Structure of *Leptin* Knockout Mice in High-Fat Diet

**DOI:** 10.3389/fmicb.2021.703929

**Published:** 2021-09-07

**Authors:** Yan Feng, Jianghao Feng, Lei Wang, Ai Meng, Siang Wei, Jie Cui, Xiongbing Hu, Lihuan Yan

**Affiliations:** ^1^College of Life Sciences, Shanxi Agricultural University, Jinzhong, China; ^2^College of Animal Science and Technology, Northwest A&F University, Xianyang, China; ^3^Shanxi Institute of Food and Drug Control, Taiyuan, China; ^4^Beijing Viewsolid Biotech Co., Ltd., Beijing, China

**Keywords:** cecal microbiota, short-chain inulin, *leptin* knockout mice, high-fat diet, probiotics, conditioned pathogenic bacteria

## Abstract

The aim of this study was to explore the effect of short-chain inulin on cecal microbiota of high-fat diet-fed *leptin* knockout mice and the different influences of cecal microbiota on wild-type and *leptin* knockout mice. A total of 18 specific pathogen-free male C57BL/6J wild-type mice and 18 C57BL/6J *leptin* knockout mice (OB/OB mice) were selected. Mice were divided into six groups according to their genotype: wild-type mice have three groups, including the normal diet group (CT), 60% high-fat diet group (CH), and 60% high fat with 10% short-chain inulin group (CHI); OB/OB mice were also divided into three groups, including the normal diet group (OT), 60% high-fat diet group (OH), and 60% high fat with 10% short-inulin group (OHI). The mice were fed for 8 weeks to analyze the diversity of cecal microbiota. The results show that compared with CH and OH, the variety of cecal microbiota was significantly reduced in CH and OH and further reduced in CHI and OHI. *Bifidobacterium* and *Lactobacillus* are the biomarkers in genus level. Dietary short-chain inulin significantly enhanced *Bifidobacterium* in OHI compared with OH (*p* < 0.01) and significantly reduced in CHI and compared with CH (*p* < 0.01). *Lactobacillus* was significantly enhanced in CHI and OHI compared with CH and OH, respectively (*p* < 0.01). *Blautia* was significantly enhanced in CH and OH compared with other groups (*p* < 0.01). Both *Escherichia-Shigella* and *Enterococcus* were significantly reduced in CHI and OHI, compared with CH and OH, respectively (*p* < 0.05). *Escherichia-Shigella* was even lower than CT and OT in CHI and OHI. Functional prediction of microbial communities showed that the abundance of amino acid sugar and nucleotide sugar metabolism pathways were significantly enhanced (*p* < 0.05) in CH and OH, and OH was significantly higher than CH (*p* < 0.05). Among the *leptin* knockout groups, PICRUSt2 function prediction showed that the fatty acid metabolism pathway significantly reduced (*p* < 0.05) in OHI and OT compared with OH. In conclusion, short-chain inulin modulated the dysbiosis induced by high-fat diet, improved probiotics growth and inhibited conditioned pathogenic bacteria, and the influences were significantly different in wild-type and *leptin* knockout mice.

## Introduction

The adoption of modern dietary habits has become a growing health concern, as it is strongly associated with obesity and related metabolic diseases by promoting inflammation and inducing gut microbiota alteration. In recent years, many studies have shown the obese are predisposed to diabetes, cardiovascular diseases, cancer, and gastrointestinal diseases such as irritable bowel syndrome (IBS) and other chronic diseases (Arumugam et al., 2011; Lin et al., 2017; Spencer et al., 2019; Thingholm et al., 2019; Lavelle and Sokol, 2020). Diet and genetic factors could both affect obesity occurrence (Wrann et al., 2012; Mohammed et al., 2018). In addition, gut microbiota have been proven to be related to body fitness and obesity formation and resistance (Dewulf et al., 2013; Barczynska et al., 2017; Shen, 2017; Lavelle and Sokol, 2020). Furthermore, high-fat diets can reduce the diversity of intestinal bacteria and meanwhile affect the homeostasis of the host (Arita and Inagaki-Ohara, 2019).

Inulin can resist obesity and improve the internal environment due to its non-digestibility that supports the colonization of probiotics and further regulates gut microbiota-related disorders (Bindels et al., 2015; Catry et al., 2018; Zhao et al., 2018). Serving as the key metabolites from inulin, short-chain fatty acids and lactate could enhance host energy metabolism by the host organism of all metabolites from inulin (Shoaib et al., 2016). It also has a fecal bulking effect like other soluble fibers, which can resist constipation and increase satiety (Anderson et al., 2009). Previous studies have proved that increasing the amount of dietary fiber can improve the microenvironment and promote the health of the host homeostasis (Holscher, 2017). Furthermore, inulin has long, medium, and short polymerization. Inulin with different degrees of polymerization has different utilization efficiencies in the intestinal tract (Ozturkoglu-Budak et al., 2019). Notably, inulin, with low polymerization between 2 and 10, is utilized much easier by microbes in the cecum than those with higher polymerization (van de Wiele et al., 2007).

*Leptin* is the essential genetic factor of obesity (de Git and Adan, 2015; Zhang and Chua, 2017). *Leptin* proteins can be used to regulate dietary intake via the nervous system while stimulating fat tissue to accelerate metabolism to maintain weight at normal levels (Pan et al., 2014). *Leptin* has a close relationship with obesity-associated heart dysfunction; it can affect cardiac contractility in ventricular myocytes (Wold et al., 2002; Dong and Ren, 2007). In addition, leptin can serve as the regulator of cardiovascular function in the physiological range, and plasma leptin levels can be used as a biomarker for cardiovascular diseases (Ren, 2004). With an abnormal level of *leptin* gene expression, obese individuals have unbalanced energy metabolism caused by resistance to leptin-relevant proteins (Ellekilde et al., 2014). Here, we hypothesized that short-chain inulin supplementation with *leptin* knockout dietary advice to increase probiotics may be an efficient way to control pathogenic bacteria and associated metabolic disorders. Overall, the present study aims to assess the effect of short-chain inulin on cecal microbiota diversity in *leptin* knockout mice fed with high-fat diets and the different effects of short-chain inulin in wild-type and *leptin* knockout mice.

## Materials and Methods

### Animal Grouping and Feed

All protocols were approved by the animal care committee of Beijing Viewsolid Biotechnology Co., Ltd. (Beijing, China). A completely randomized design was used in this study. The short-chain inulin was purchased from DPO Beijing Co., Ltd. (Beijing, China). The regular diet, 60% high-fat diet, and 60% high-fat diet with 10% short-chain inulin were customized by Beijing Keao Xieli Feed Co., Ltd. (Beijing, China). All diets underwent irradiation sterilization after production and autoclaved sterilization before use. This research used 36 specific pathogen-free (SPF) 6-week-old male C57BL/6J mice, with half wild-type and half *leptin* gene knockout. All mice were supplied by Beijing Viewsolid Biotech Co., Ltd. (Beijing, China). All mice were fed in SPF cages from Beijing Viewsolid Biotech Co., Ltd., with free intake of diets and water, as an overall process for 8 weeks. Wild-type mice were divided into three groups: normal diet (CT), 60% high-fat diet (CH), and 60% high fat with 10% short-chain inulin (CHI); *leptin* knockout mice were also divided into three groups: normal diet (OT), 60% high-fat diet (OH), and 60% high fat with 10% short-chain inulin (OHI).

### Sample Collection

All 36 mice were killed by cervical dislocation after 8 weeks of feeding trial. Cecum luminal contents were collected into two frozen tubes and stored at –80°C for DNA extraction and 16S rDNA analysis.

### Diversity of Intestinal Microbiota

Microbiota diversity and the evolutionary variance of cecum in all treatments were analyzed. Microbial community genomic DNA was extracted from 36 cecum samples using the E.Z.N.A.^®^ soil DNA Kit (Omega Bio-tek, Norcross, GA, United States) according to manufacturer’s instructions. The DNA extract was checked on 1% agarose gel, and DNA concentration and purity were determined using the NanoDrop 2000 UV-vis spectrophotometer (Thermo Scientific, Wilmington, DE, United States). After the sampling of contents in cecum segments from all treatments, the bacterial genomic DNA of these contents was extracted using the universal primer pair 338F (5′-ACT CCT ACG GG AGG CAG CAG-3′) and 806R (5′-GGA CTA CHV GGG TWT CTAAT-3′), and were used to amplify the V3–V4 regions of genomic16S rDNA, following the examination and collection of PCR products by 2% agarose gel electrophoresis. The quantitative detection was conducted using the Quanti FluorTM-ST blue fluorescence quantitative system (Promega, Madison, WI, United States). The sequences of 16S rDNA fragment were obtained and summed up with the MiSeq platform (Illumina, San Diego, CA, United States).

### Statistical Analysis

The raw 16S rRNA gene sequencing reads were demultiplexed, quality-filtered using FASTP version 0.20.0, and merged using FLASH version 1.2.7 with the following criteria: the 300 bp reads were truncated at any site receiving an average quality score of < 20 over a 50 bp sliding window; the truncated reads shorter than 50 bp were discarded; reads containing ambiguous characters were also discarded; only overlapping sequences longer than 10 bp were assembled according to their overlapped sequence. The maximum mismatch ratio of the overlap region is 0.2. Reads that could not be assembled were discarded. Samples were distinguished according to the barcodes and primers, and the sequence direction was adjusted. Exact barcode matching was performed, yielding two nucleotide mismatches in primer matching.

Operational taxonomic units (OTUs) with a 97% similarity cutoff were clustered using UPARSE version 7.1, and chimeric sequences were identified and removed. The taxonomy of each OTU representative sequence was analyzed using RDP Classifier version 2.2 against the 16S rRNA database (Silva v138), using a confidence threshold of 0.7.

After the preliminary arrangement of data, SPSS 24.0 (Chicago, IL, United States) was used to analyze the dissimilarity among treatments. Duncan multiple comparative tests proceeded for the data with significant imparity. Differences among dietary treatments were analyzed by the Kruskal–Wallis *H* test. Values of *p* < 0.05 were considered statistically remarkable. Alpha analysis was performed by using R package Mothur version 1.30.2. PICRUSt version 1.1.0 was used to predict pathways based on the SILVA, UNITE, EggNOG, KEGG, and MetaCyc data sets.

## Results

### Weight

**FIGURE 1:**
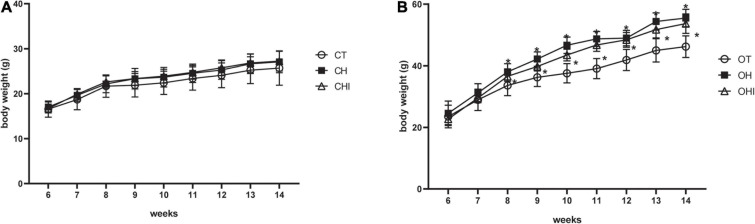
Effect of high-fat diet and inulin supplementation on body weight. **(A)** Effect of normal diet, high-fat diet, and high fat with inulin diet on wild-type mice. **(B)** Effect of normal diet, high-fat diet, and high fat with inulin diet on leptin knockout mice. “*” indicates a significant difference compared with OT (*p* < 0.05).

In the present study, the 6-week-old mice were fed with normal diet, 60% high-fat diet, or high-fat diet with 10% inulin diet, respectively. The test was terminated at 14 weeks. The weight gain is shown in Figure 1. Among the wild-type groups, there were no significant differences (Figure 1A). Among the *leptin* knockout groups, the weight was significantly enhanced in OH and OHI when compared to OT (*p* < 0.05). No difference was observed between OHI and OH (Figure 1B).

### Abundance of Cecal Microbiota

The results of alpha diversity analysis for cecal microbiota in different treatments are listed in Table 1. In the three wild-type groups, compared with CT, the Sobs index, Chao1 index, and Shannon index of CH and CHI significantly reduced (*p* < 0.01), The Simpson index of CHI was significantly enhanced (*p* < 0.01). In addition, among the *leptin* gene knockout groups, the Sobs index, Chao1 index, Shannon index, and Simpson index were significantly reduced in OH and OHI when compared to OT (*p* < 0.01). In different genotypes, only the normal diet CT and OT have a significant difference. The Sobs index and Chao1 index were significantly reduced in OT compared with CT (*p* < 0.01).

**TABLE 1:** Alpha diversity of cecal microbiota.

**Index**	**CT**	**CH**	**CHI**	**OT**	**OH**	**OHI**	***P*-value**
Sobs	434.5032.40^A^	227.1720.06^C^	198.1722.80^C^	356.6730.22^B^	224.6732.94^C^	194.3343.70^C^	<0.001
Shannon	4.250.21^A^	2.920.39^BC^	2.420.45^C^	4.160.18^A^	3.16031^B^	2.660.35^BC^	<0.001
Simpson	0.040.01^B^	0.130.08^AB^	0.260.13^A^	0.030.01^B^	0.100.03^A^	0.150.05^A^	<0.001
Chao1	488.0044.30^A^	293.5837.46^C^	252.1735.10^C^	405.5432.92^B^	278.7244.91^C^	264.1581.62^C^	<0.001

*The results are shown with mean ± standard error and P values are analyzed with student’s t-test. Sobs index shows the observed richness. Chao1 index reflect the abundance. Shannon index is positively and Simpson index is negatively related to the richness and uniformity of microbiota in samples. ^*A,B,C*^ means in the same row with no common superscripts are significantly different (*p* < 0.01).*

**FIGURE 2:**
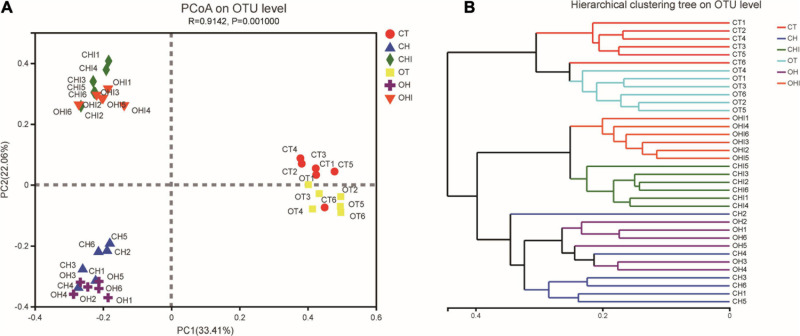
The sectionalization of cecal microbiota. **(A)** Principal coordinate analysis (PCoA) of 16S sequences from 36 cecum content samples of six treatments based on Weighted Unifrac. **(B)** Hierarchical cluster tree of cecal microbiota. The length between the branches represents the distance between the samples, and different groups can be presented in different colors.

The PCoA (Figure 2A) and hierarchical cluster tree (Figure 2B) based on Unweighted UniFrac distance were used to evaluate variety in the microbiota composition of cecum. Each group had a high degree of polymerization; for CT, OT and OH, CH, the distribution areas of these treatments overlapped. There were short distances between the same-type diet groups, and long distances between the normal diet and the other two diet groups. The hierarchical cluster tree showed that normal diet, high-fat diet, and high fat with 10% inulin were divided into three distinct microbiomes. Each microbiome has two genotype groups, and each group has a close branch length between their own samples except the microbiome of the high-fat diet.

**FIGURE 3:**
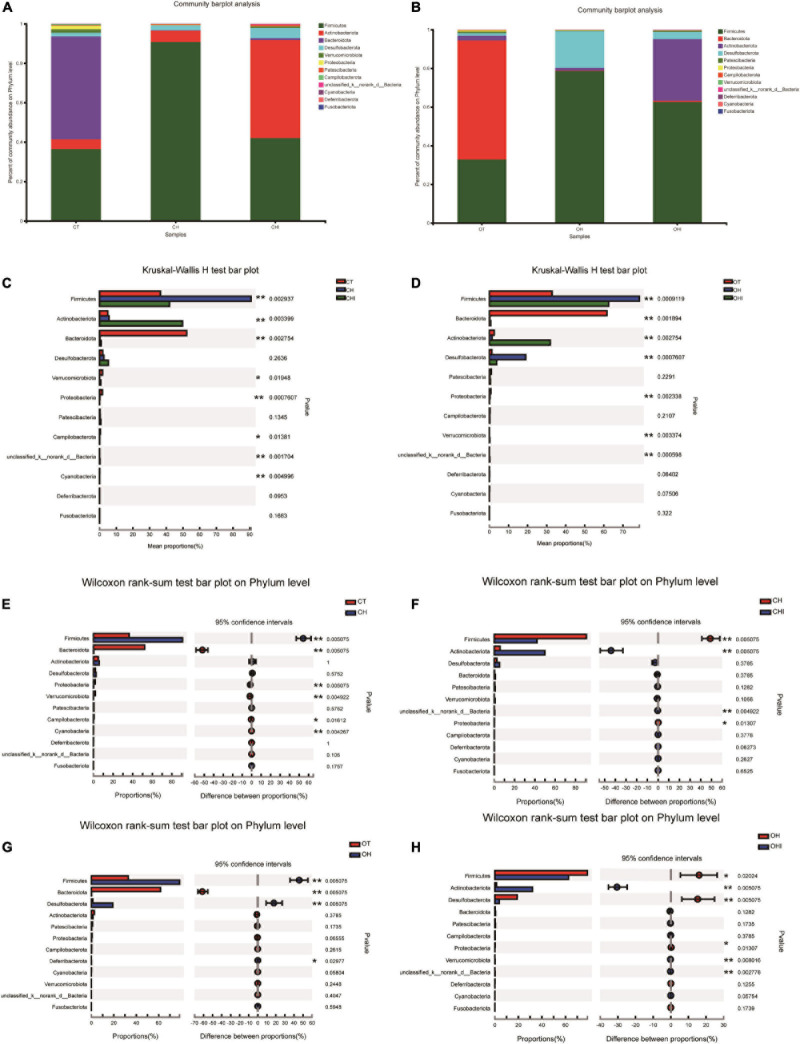
Relative abundance of cecal microbiota in phylum level. **(A)** Relative abundance of cecal microbiota for CT, CH, and CHI groups on phylum level. **(B)** Relative abundance of cecal microbiota for OT, OH, and OHI groups on phylum level. **(C)** The cecal microbiota showed differential abundance at the phylum level between the CT, CH, and CHI groups. **(D)** The cecal microbiota showed differential abundance at the phylum level between the OT, OH, and OHI groups. **(E)** The cecal microbiota showed differential abundance at the phylum level between the CT and CH groups according to *t*-tests. **(F)** The cecal microbiota showed differential abundance at the phylum level between the CH and CHI groups according to *t*-tests. **(G)** The cecal microbiota showed differential abundance at the phylum level between the OT and OH groups according to *t*-tests. **(H)** The cecal microbiota showed differential abundance at the phylum level between the OH and OHI groups according to *t*-tests. *Indicated there was a significant difference compared with OT (*p* < 0.05), ** indicated there was a significant difference compared with OT (*p* < 0.01).

**FIGURE 4:**
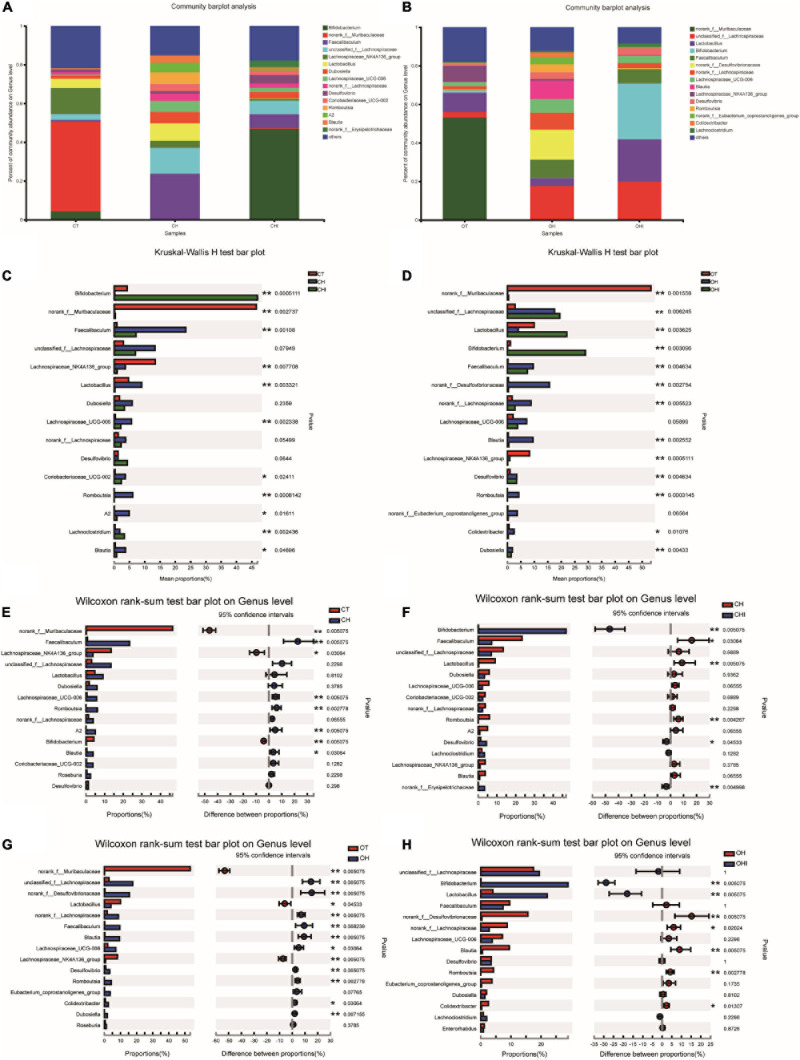
Top 15 microbiota relative abundance of cecal microbiota in genus level. **(A)** Relative abundance of cecal microbiota for CT, CH, and CHI groups on genus level. **(B)** Relative abundance of cecal microbiota for OT, OH, and OHI groups on genus level. **(C)** The cecal microbiota showed differential abundance at the genus level between the CT, CH, and CHI groups. **(D)** The cecal microbiota showed differential abundance at the genus level between the OT, OH, and OHI groups. **(E)** The cecal microbiota showed differential abundance at the genus level between the CT and CH groups according to *t*-tests. **(F)** The cecal microbiota showed differential abundance at the genus level between the CH and CHI groups according to *t*-tests. **(G)** The cecal microbiota showed differential abundance at the genus level between the OT and OH groups according to *t*-tests. **(H)** The cecal microbiota showed differential abundance at the genus level between the OH and OHI groups according to *t*-tests. *Indicated there was a significant difference compared with OT (*p* < 0.05), ** indicated there was a significant difference compared with OT (*p* < 0.01).

**FIGURE 5:**
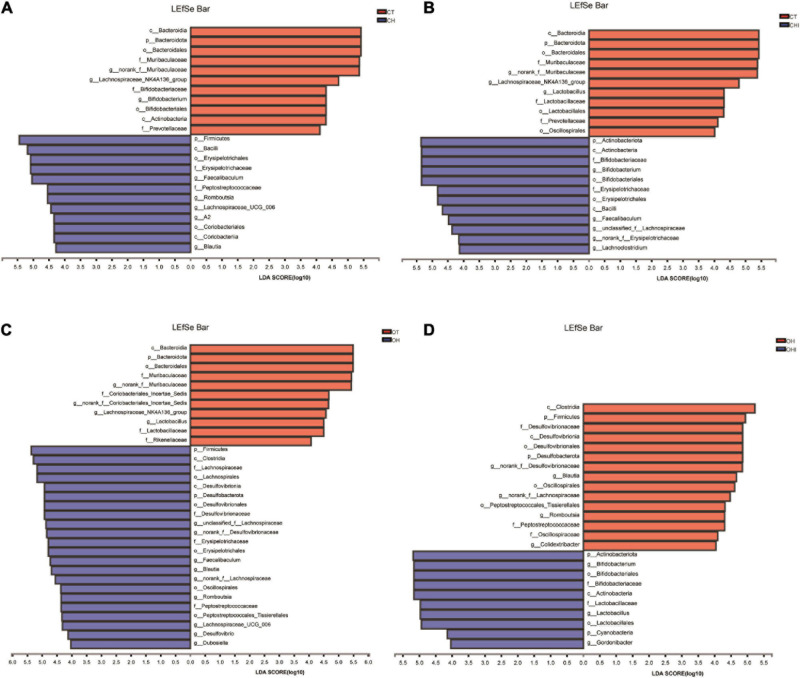
Linear discriminant analysis (LDA) compared the alterations in cecum microbiome according to the diet and genotype. **(A)** The effect of species abundance on the difference in CT and CH. **(B)** The effect of species abundance on the difference in CH and CHI. **(C)** The effect of species abundance on the difference in OT and OH. **(D)** The effect of species abundance on the difference in OH and OHI.

After the analysis of the microbiota structure in the cecum, the relative abundance of phylum levels and genus levels are shown in Figures 3, 4. The linear discriminant analysis effect size (LEfSe) multilevel discriminant analysis results of cecum microbiota are shown in Figure 5. The multi-group comparison strategy of all-again-all was used to tighten the definitions of the differences. This analysis can show that the microbiota have an important effect on the difference from phylum to genus. It can further find that microbiota are important to the difference. The differential abundance of the genera was analyzed using the Kruskal–Wallis *H* test. At the phylum level, Firmicutes (36.4–90.7%), Actinobacteriota (4.9–49.7%), and Bacteroidota (0.28–52.1%) are the dominant phyla across the CT, CH, and CHI groups (Figure 3A). Firmicutes (36.4–90.7%), Actinobacteriota (4.9–49.7%), Bacteroidota (0.28–52.1%), and Desulfobacterota (1.2–19.0%) are the dominant phyla across the OT, OH, and OHI groups (Figure 3B). In the wild-type groups, high-fat diet significantly increased the abundance of Bacteroidota (*p* = 0.0029). Short-chain inulin significantly increased the abundance of Actinobacteriota (*p* = 0.0033) and altered the relative the abundance of Bacteroidota (*p* = 0.0029) (Figure 3C). High-fat diet significantly increased the abundance of Firmicutes and reduced Bacteroidota (*p* = 0.0050), Proteobacteria (*p* = 0.0050), Verrucomicrobiota (*p* = 0.0049), Cyanobacteria (*p* = 0.0043), and Campilobacterota (*p* = 0.0161) in CH compared with CT (Figure 3E). However, short-chain inulin enhanced the abundance of Actinobacteriota (*p* = 0.0050) and unclassified_k__norank_d__Bacteria (*p* = 0.0049), and reduced the abundance of Firmicutes (*p* = 0.0050) and Proteobacteria (*p* = 0.0130) in CHI compared with CH (Figure 3F). In the *leptin* knockout groups, high-fat diet significantly increased the abundance of Bacteroidota (*p* = 0.0009) and Desulfobacterota (*p* = 0.0007). Short-chain inulin also increased the abundance of Actinobacteriota (*p* = 0.0027) and reduced the abundance of Bacreroidota (*p* = 0.0009) (Figure 3D). Compared with OT, high-fat diet reduced the abundance of Bacteroidota (*p* = 0.0050), and enhanced the abundance of Desulfobacterota (*p* = 0.0050), Deferribacterota (*p* = 0.0297), and Firmicutes (*p* = 0.0050) in OH (Figure 3G). In OHI, short-chain inulin intake enhanced the abundance of Verrucomicrobiota (*p* = 0.0080), unclassified_k__norank_d__Bacteria (*p* = 0.0027), and Actinobacteriota (*p* = 0.0050) and reduced the abundance of Firmicutes (*p* = 0.0202), Desulfobacterota (*p* = 0.0050), and Proteobacteria (*p* = 0.0130) (Figure 3H).

**FIGURE 6:**
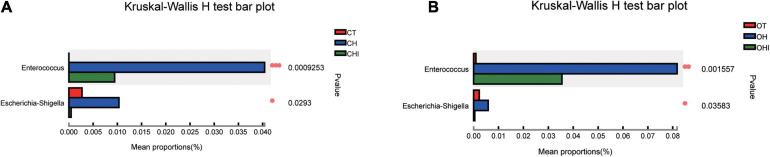
Conditioned pathogenic bacteria. Relative abundance of cecal microbiota at the genus level. **(A)** Conditioned pathogenic bacteria showed differential abundance at the genus level among the CT, CH, and CHI groups according to one-way ANOVA. **(B)** Conditioned pathogenic bacteria showed differential abundance at the genus level among the OT, OH, and OHI groups according to one-way ANOVA. * indicates a significant difference compared with OT (*p* < 0.05), ** indicates a significant difference compared with OT (*p* < 0.01).

A total of 196 bacterial genera were identified. In the three wild-type groups the top 15 genera in the cecal microbiota were identified, accounting for 82.1–91.7% among the wild-type groups (Figure 4A) and accounting for 78.3–84.9% among the *leptin* knockout groups (Figure 4B). *norank_f_Muribaculaceae* is the dominant genus in CT. The abundance of *norank_f_Muribaculaceae* (*p* = 0.0005) and *Lachnospiraceae_NK4A136_group* (*p* = 0.0077) was significantly higher than in CH and CHI. High-fat diet increased other genera which were less in CT and CHI. *Faecalibaculum* (*p* = 0.0010), *Lactobacillus* (*p* = 0.0033), *Romboutsia* (*p* = 0.0008), and *Lachnospiraceae_UCG-006* (*p* = 0.0023) were significantly increased in CH. *Bifidobacterium* was the dominant genus in CHI, and the abundance was significantly higher than CT and CH (Figure 4C). Compared with CT, high-fat diet enhanced the abundance of *Faecalibaculum* (*p* = 0.0050), *Lachnospiraceae_UCG-006* (*p* = 0.0050), *Romboutsia* (*p* = 0.0027), *A2* (*p* = 0.0050), and *Blautia* (*p* = 0.0306) and reduced the abundance of *norank_f__Muribaculaceae* (*p* = 0.0050), *Lachnospiraceae_NK4A136_group* (*p* = 0.0306), and *Bifidobacterium* (*p* = 0.0050) (Figure 4E). In CHI, short-chain inulin reduced the abundance of *Faecalibaculum* (*p* = 0.0306), *Lactobacillus* (*p* = 0.0050), and *Romboutsia* (*p* = 0.0042), and enhanced the abundance of *Bifidobacterium* (*p* = 0.0050), *norank_f__Erysipelotrichaceae* (*p* = 0.0049), and *Desulfovibrio* (*p* = 0.0453) compared with CH (Figure 4F). In the *leptin* knockout groups, *norank_f_Muribaculaceae* was also the dominant genus in OT, and the abundance of *norank_f_Muribaculaceae* (*p* = 0.0015) and *Lachnospiraceae_NK4A136_group* (*p* = 0.0005) is significantly higher than in OH and OHI. High-fat diet increased the abundance of *unclassified_f__Lachnospiraceae* (*p* = 0.0062), *Faecalibaculum* (*p* = 0.0046), *norank_f__Desulfovibrionaceae* (*p* = 0.0027), *Blautia* (*p* = 0.0025), and *Romboutsia* (*p* = 0.0003). Short-chain inulin increased the abundance of *Lactobacillus* (*p* = 0.0036) and *Bifidobacterium* (*p* = 0.0030) (Figure 4D). High-fat diet increased the abundance of *unclassified_f__Lachnospiraceae* (*p* = 0.0050), *norank_f__Desulfovibrionaceae* (*p* = 0.0050), *Lactobacillus*, *norank_f__Lachnospiraceae* (*p* = 0.0050), *Faecalibaculum* (*p* = 0.0080), *Blautia* (*p* = 0.0050), *Lachnospiraceae_UCG-006* (*p* = 0.0306), *Desulfovibrio* (*p* = 0.0050), *Romboutsia* (*p* = 0.0027), *Colidextribacter* (*p* = 0.0306), and *Dubosiella* (*p* = 0.0071), and reduced the abundance of *norank_f__Muribaculaceae* (*p* = 0.0050) and *Lachnospiraceae_NK4A136_group* (*p* = 0.0050) in OH compared with OT (Figure 4G). After the addition of short-chain inulin, *Bifidobacterium* (*p* = 0.0050) and *Lactobacillus* (*p* = 0.0050) were significantly enhanced, and *norank_f__Desulfovibrionaceae* (*p* = 0.0050), *norank_f__Lachnospiraceae* (*p* = 0.0202), *Blautia* (*p* = 0.0050), *Romboutsia* (*p* = +−0.0027), and *Colidextribacter* (p 9 0.0130) were significantly reduced in OHI compared with OH (Figure 4H). In addition, we also found that high-fat diet increased the abundance of the conditioned pathogenic bacteria *Escherichia-Shigella* and *Enterococcus* in cecum. Short-chain inulin can significantly reduce the abundance of *Escherichia-Shigella* (*p* = 0.0009) and *Enterococcus* (*p* = 0.0293) in CHI (Figure 6A). In OHI, short-chain inulin also has the same effect; the abundance of *Escherichia-Shigella* (*p* = 0.0015) and *Enterococcus* (*p* = 0.0358) was reduced in OHI (Figure 6B).

### Network Interaction of Cecal Microbiota

**FIGURE 7:**
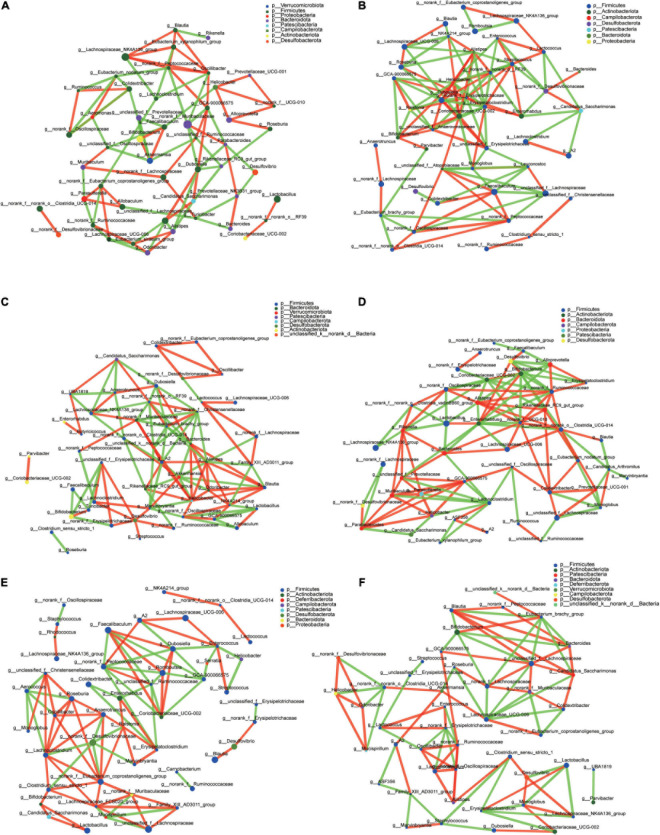
Microbiota correlation network of different groups. **(A)** Correlation network of CT. **(B)** Correlation network of CH. **(C)** Correlation network of CHI. **(D)** Correlation network of OT. **(E)** Correlation network of OH. **(F)** Correlation network of OHI. The red line indicates a positive correlation (*p* > 0.06), and the green line indicates a negative correlation (*p* < –0.06).

The top 50 genera correlation is shown in Figure 7. Using spearman rank to value the correlation coefficient, only the absolute value higher than 0.6 can be shown. *Blautia* in each group had a different correlation. In CT it had a positive correlation with *Lachnospiraceae_NK4A136_group*, *Eubacterium_xylanophilum_group*, and *Oscillibacter*, and a negative correlation with *Rikenella* (Figure 7A). In CH, there was a positive correlation with *Lachnospiraceae_UCG-006* and negative correlation with *NK4A214_group* (Figure 7B). In CHI, there were five genera, *Alistipes*, *Bacteroides*, *Odoribacter*, *Helicobacter*, and NK4A214_group, which had positive a correlation with *Blautia*; *Family_XIII_AD3011_group* and *Akkermansia* had a negative correlation with *Blautia* (Figure 7C). In the three *leptin* knockout groups, the associate genera with *Blautia* were different. *Clostridia_UCG-014* had a positive correlation with *Blautia*, and *Candidatus_Arthromitus* had a negative correlation with *Blautia* in OT, respectively (Figure 7D). In OH, only *Desulfovibrio* had a positive correlation with *Blautia* (Figure 7E). In OHI, *norank_f__Peptococcaceae*, *Bacteroides*, *unclassified_f__Lachnospiraceae*, and *Candidatus_ Saccharimonas* had a positive correlation with *Blautia*, and *Eubacterium_brachy_group*, *Bifidobacterium* had a negative correlation with *Blautia* (Figure 7F).

### PICRUSt2 Function Prediction

**FIGURE 8:**
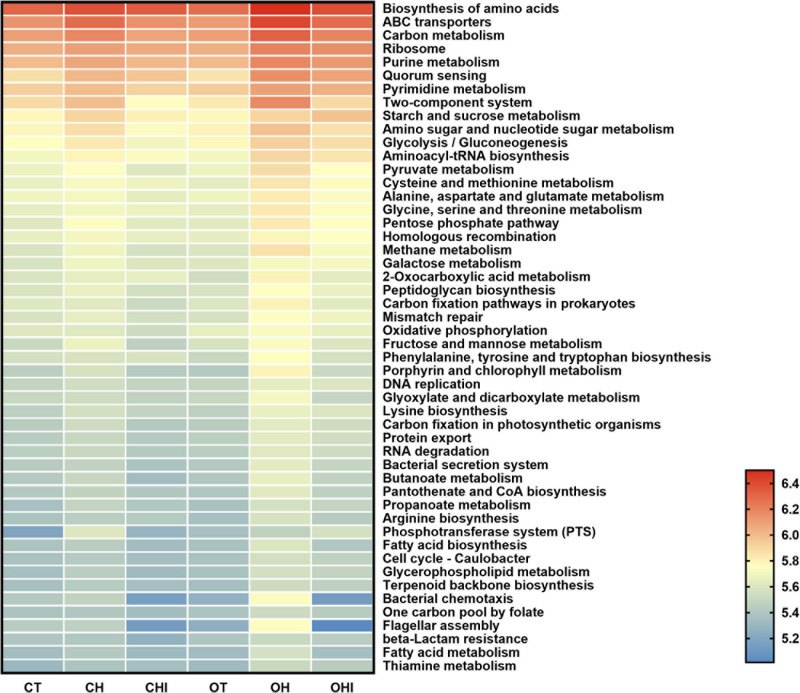
Top 50 pathway into level 3 KEGG as predicted by PICRUSt2.

PICRUSt2 function prediction was performed by comparing the microbiota function with the sequencing information and KEGG database. The results were shown in Figure 8. The comparison of enzymes and metabolic pathways were related to the fatty acid metabolism pathway. Glucose metabolism and lipid metabolism found that the abundance of amino acid sugar and nucleotide sugar metabolism pathways were enhanced (*p* < 0.05) in the high-fat diet compared with the normal diet, and OH was higher than CH (*p* < 0.05) Short- chain inulin reduced these pathways in CHI and OHI.

## Discussion

There are numerous studies about inulin, whereas few of them focused on the effect of low-polymerization inulin on gut microbiota. Our study focused on the effect of short-chain inulin on cecal microbiota, and found that its effect varies between wild-type and *leptin* knockout mice. In accordance with the previous studies, the weight of mice is significantly increased in *leptin* knockout mice when compared with wild-type mice, and the *leptin* knockout mice showed an obese character (Halaas et al., 1995; Bachman et al., 2002). Furthermore, different dietary treatments had effects on the weight of *leptin* knockout mice; in the current study, the weight of OHI and OH mice were significantly increased when compared with OT. However, there is no significant difference among the wild-type groups. The results demonstrated that wild-type mice are more resistant to dietary changes than *leptin* knockout mice. It has been reported that some mice develop obesity more easily and some mice can resist obesity and keep slim in the dietary obese mice model (Bardova et al., 2016). Gut microbiota dysbiosis is also an important factor of obesity (Landman and Quevrain, 2016). Wild-type mice have a more diverse microbiota than *leptin* knockout mice; This can help mice resist obesity.

As shown in the PCoA of cecal microbiota, there were three groups of all mice according to different diets, and no overlap between each group. Specifically, it has been shown that diet can significantly change the structure of cecal microbiota. The hierarchical cluster tree showed that in CT, OT, CHI, and OHI, samples had a close relative in their own group, but in CH and OH, the distance of mice was mass, and there was no obvious arrangement according to their genotype. This result indicated that genotype has a significant influence on microbiota structure in a different diet, but in a high-fat diet, this influence is weaker. Adding short-chain inulin can restore the cecal microbiota structure to a certain extent. However, the abundance of cecal microbiota has a further reduction in high fat supplemented with short-chain inulin, probably because short-chain inulin is easy decompose in the cecum by some bacteria due to its low degree of polymerization and affects the cecum environment to inhibit the growth of other bacteria (Song et al., 2019). Similar to our results, previous studies have shown that inulin that had a different degrees of polymerization is resolved in intestinal and had different effects on microbiota (Yasuda et al., 2007). Dietary fiber intake could not always enhance the microbiota abundance. Inulin with low polymerization degree has a negative effect on microbial diversity. Some research also showed that excessive intake of dietary fiber can have a negative effect on health and increases the risk of liver cancer (Singh et al., 2018).

A review of the research has shown that intestinal microbiota are diverse in different genotypes, and diet intake also has an influence on intestinal microbiota; furthermore, high-fat diet can also reduce the diversity of intestinal microbiota (David et al., 2014; Barczynska et al., 2017; Ferrario et al., 2017; Bai et al., 2018; Zhao et al., 2018; Kumar et al., 2020). In this study, high-fat diet reduced the diversity of cecal microbiota. The abundance changes in each group showed that short-chain inulin can enhance the abundance of *Bifidobacterium*. This result is consistent with previous studies (Vandeputte et al., 2017). Some have demonstrated that *Lactobacillus* has a potential obesity resistance (Mishra et al., 2015; Nova et al., 2016), but in wild-type groups, short-chain inulin reduces the abundance of *Lactobacillus*. In *leptin* knockout mice, short-chain inulin has a different influence on cecal microbiota. Briefly, high-fat diet increased the abundance of *Lactobacillus* and *Blautia*. However, short-chain inulin reduced the abundance *Lactobacillus* and *Bifidobacterium*. In *leptin* knockout groups short-chain inulin has a positive effect on the two traditional probiotics; the body weight of CHI was a little lower than CH. This result is same as the research that *Lachnospiraceae* has a potential obesity resistance effect (Niness, 1999), but this condition has been shown only in *leptin* knockout groups. In addition, the latest research point that *Blautia* was closely related to obesity and it significantly reduced in obese children (Benitez-Paez et al., 2020; Liu et al., 2021). However, our study received a totally inverse result that *Blautia* was significantly enhanced in high-fat diet groups. Our other experiments also showed the same results. *Blautia* abundance depends more on dietary intake, and the reason of *Blautia* abundance differing from previous research needs further research. Further network correlation analysis showed that the relative microbiota of *Blautia* in high-fat diet groups were low, and in high-fat diet with short-chain inulin group it was high. All relative microbiota belong to *Verrucomicrobiota*, *Firmicutes*, and *Bacteroidota*. Considering the change of *Blautia* abundance and the microbiota diversity in each group, we can speculate that although in high-fat diet the abundance of *Blautia* is high compared with other diet groups, the contribution to microbiota diversity is less. After adding short-chain inulin the abundance of *Blautia* is decreased, and the influence of *Blautia* on microbiota diversity is increased. However, the mechanism of *Blautia* abundance change needs future research. All results in two genotype mice showed a different reaction to short-chain inulin. *Lactobacillus* and *Bifidobacterium* are two major genera which have many probiotics species. The change of these two genera in cecum was not same. As for *Blautia* our results were different from other studies, so the reason needs future verification. Our study also shows that short-chain inulin can reduce the abundance of some conditioned pathogenic bacteria. The change of *Enterococcus* and *Escherichia-Shigella* among each group indicates that high-fat diet has a negative effect on normal cecal microbiota. It can increase conditioned pathogenic bacteria and enhance the risk of cecal disease. Short-chain inulin can significantly reverse the conditioned pathogenic bacteria increase caused by a high-fat diet, modulate cecal microbiota structure, improve the abundance of probiotics, and inhibit the growth of some conditioned pathogenic bacteria.

In addition, the PICRUSt2 forecast analysis shows that the effects of genotype differences on metabolic pathways are significant. This analysis is based on metagenomic sequencing, and it can provide a guide to future research. Amino acid synthesis, sugar, and energy metabolism pathways were improved in the high-fat feeding group compared with the other two kinds of feeding groups, and the phenomenon is more obvious in the *leptin* knockout mice. The metabolic pathway in the OH mice was significantly higher than the two other groups among the same genotype. These results indicate that high-fat diet has an impact on the energy metabolism pathway, and the effect is more significant in case of *leptin* knockout, which can further enhance the activity of the energy metabolism pathway. In addition, the glucose metabolism pathway of *leptin* knockout mice is significantly higher in the high-fat and high-fat plus inulin groups than in the wild-type mice. These results indicate that *leptin* knockout significantly increases the metabolic pathway of mice in the case of dietary and dietary extremes, and *leptin* knockout led to further improvements in metabolic capacity in obese mice on a high-fat diet, as well as some changes in metabolic pathways. It has been reported that inulin can alleviate metabolism disorders in *leptin* knockout mice by partially restoring leptin-related pathways mediated by gut microbiota through AMPK pathway fatty acid oxidation in peripheral tissues (Backhed et al., 2007; Song et al., 2019). There are no significant differences between the high fat with short-chain inulin and the normal diet, which indicates that the addition of short inulin could restore the metabolic function of individuals to a certain extent and make the metabolic pathway closer to the normal state. The effects of genotype and feeding composition on metabolic pathways still need further experimental verification.

## Conclusion

In high-fat diet, the addition of short-chain inulin can reduce cecal microbiota diversity. The effect of short-chain inulin on cecal microbiota abundance was different in wild-type and *leptin* knockout mice. Short-chain inulin can promote probiotics and inhibit conditioned pathogenic bacteria growth in both genotypes and can enhance *Lactobacillus* and *Bifidobacterium* and reduce *Blautia*, *Enterococcus*, and *Escherichia-Shigella* growth. In addition, short-chain inulin can change some lipid and amino acid metabolic pathways and modulate these pathways in response to high-fat diet.

## Data Availability Statement

The datasets presented in this study can be found in online repositories. The names of the repository/repositories and accession number(s) can be found below: https://www.ncbi.nlm.nih.gov/, PRJNA725111.

## Ethics Statement

The animal study was reviewed and approved by the Beijing Viewsolid Biotech Company Limited (20200046).

## Author Contributions

YF and JF: conceptualization. JF and LW: methodology. JF, AM, and LY: investigation. JF and SW: formal analysis. JF: writing – original draft. YF, LW, and JC: writing – review and editing. XH: resources. All authors have read and agreed to the published version of the manuscript.

## Conflict of Interest

XH was employed at Beijing Viewsolid Biotech Co., Ltd. The remaining authors declare that the research was conducted in the absence of any commercial or financial relationships that could be construed as a potential conflict of interest.

## Publisher’s Note

All claims expressed in this article are solely those of the authors and do not necessarily represent those of their affiliated organizations, or those of the publisher, the editors and the reviewers. Any product that may be evaluated in this article, or claim that may be made by its manufacturer, is not guaranteed or endorsed by the publisher.
